# Double left ventricular pacing following accidental malpositioning of the right ventricular electrode during implantation of a cardiac resynchronization therapy device

**DOI:** 10.1186/1749-8090-8-162

**Published:** 2013-06-27

**Authors:** Ruediger Dissmann, Udo Wolthoff, Markus Zabel

**Affiliations:** 1 Department Cardiology/Nephrology, Klinikum Bremerhaven Reinkenheide, Postbrookstr. 103, D-27574, Bremerhaven, Germany; 2 Division of Cardiology and Pneumology, Georg August University Goettingen, Robert-Koch-Str. 40, D-37099, Goettingen, Germany

**Keywords:** Misplaced Leads, Malpositioned Leads, Implantable Cardioverter-defibrillator, Pacemaker, Cardiac Resynchronzation Therapy, Complications

## Abstract

Accidental malpositioning of a right ventricular (RV) electrode has not been previously reported in the context of cardiac resynchronization therapy (CRT).

The case of a 75-year old male patient with dilative cardiomyopathy, left ventricular (LV) ejection fraction 23%, New York Heart Association functional heart failure status stage III, left bundle branch block (LBBB) with QRS width of 136 ms, and misplacement of the RV lead to the LV apex during implantation of a CRT defibrillator is described.

Following unremarkable implantation, routine interrogation of the CRT device on the first day after the implantation revealed uneventful technical findings. The 12-lead surface electrocardiogram (ECG) showed biventricular stimulation featuring a narrow QRS complex with incomplete right bundle branch block (RBBB) and R>S in V1. The biplane postoperative chest X-ray was graded normal. On routine follow-up one month later, a transthoracic echocardiogram revealed an increased ejection fraction of 51% but the RV lead was placed in the LV apex. An additional transesophageal echocardiogram exhibited an Eustachian valve guiding the lead via the patent foramen ovale through the mitral valve into the LV apex. Operative revision was scheduled and the active fixation lead was uneventful removed from the LV. A new electrode was inserted and placed in the RV apex.

Accidental malplacement of the RV electrode to the LV may be difficult to diagnose in the context of CRT patients as a stimulated biventricular ECG with incomplete RBBB appearance is expected in this situation. Careful analysis of lateral radiographic views during the operation is important to ensure correct lead positioning. As timely revision is the preferred procedure, early routine transthoracic echocardiography may be considered for detection of malplacement.

## Background

In most cases, implantation of pacemaker or defibrillator leads is a straightforward routine procedure. In selected patients difficulties may be caused by congenital variants [1], angiologic abnormalities [2] or misplacement of leads into the arterial circulation [3-6]. In the literature, there are less than 50 case reports describing an incidental malplacement of right ventricular (RV) electrodes into the left ventricle (LV) [4,5]. Usually, the malplacement can be detected by the postprocedure twelve-lead electrocardiogram (ECG) exhibiting an unexpected right bundle branch block (RBBB) pattern instead of the usual left bundle branch (LBBB) appearance [4]. After successful cardiac resynchronization therapy (CRT) procedures, however, combined pacing of RV and LV usually results in a RBBB configuration and a misplaced RV-lead may therefore be missed. To our best knowledge, this problem has not been reported by case reports or recent review articles [4].

## Case presentation

**Figure 1:**
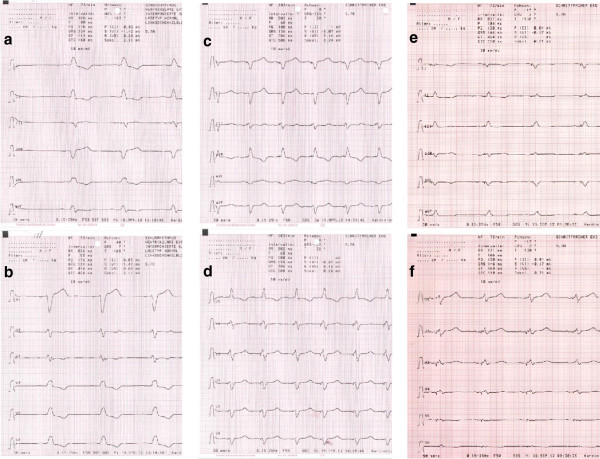
**ECC recordings before CRT implantation showed a wide QRS due to LBBB (1 a-b); after CRT implantation simultaneous stimulation of the presumed RV electrode misplaced in the left ventricle and the CS electrode resulted in a narrow QRS and positive R-waves in V1 (1 c-d); after revision of the RV lead simultaneous stimulation in the RV and the CS (1 e-f).** Although the ECGs before and after revision show differences, the principle finding of a narrow QRS and a positive R-Wave in V1 is present in both recordings.

**Figure 2:**
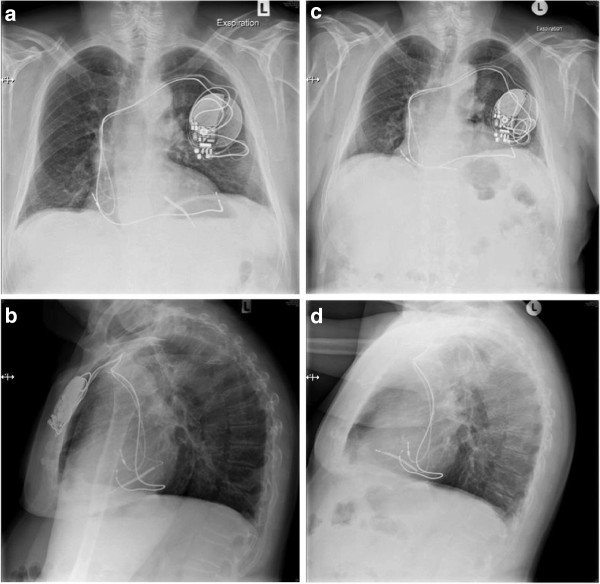
**Chest x-ray in the anterior and lateral view after the original CRT-ICD implantation (2 a-b) and after revision of the ICD lead (2 c-d).** Atrial and CS electrodes remained unchanged. During revision the malplaced ICD electrode was removed and replaced in the RV. The chest x-ray after first implant was graded normal by routine judgement. In retrospective, the curve at the atrial level and the posterior orientation of the ICD lead may indicate passage of the foramen ovale and misplacement in the LV.

A 75-year-old caucasian male patient with a diagnosis of dilative cardiomyopathy (echocardiographic biplane ejection fraction (EF) 23%) had sinus rhythm with left LBBB ECG (Figure 1a-b) (QRS with 136 ms) and a profound dyssynergic contraction profile. There was a history of Diabetes Typ II, hypertension and mild renal insufficiency (serum creatinine 1.63 mg/dl). Significant coronary artery disease had been excluded by angiography. Currently, he suffered from New York Heart Association (NYHA) III heart failure status and was admitted for implantation of a CRT – implantable cardioverter defibrillator (ICD). The operative procedure was unremarkable, although some difficulties advancing the right electrode into the right ventricle were reported. Chest X-ray after implantation (Figure 2a-b) excluded pneumothorax and electrodes appeared well placed. The patient was discharged one day after the procedure when the pacing ECG showed a narrowed QRS complex (Figure 1c-d) with optimal values of all three electrodes (Table 1).

**Table 1:** **CRT**-**D system values one day and four weeks** (**in parentheses**) **after the implantation**

**Boston scientific punctua CRT-D (REF. P052)**	**Right atrium**	**Right ventricle**	**Left ventricle/ Coronary sinus**
Electrodes	Flextend II 4096, Fa. Boston scientific	Reliance SG 0293, Fa. Boston scientific	Acuity steerable 4555, Fa. Boston S.
Amplitude	5.4 (3.7) Volt	10.4 (11.0) Volt	17.7 (25.0) Volt
Impedance	486 (475) Ohm	390 (359) Ohm	973 (811) Ohm
Stimulation threshhold	0.5 (0.7) mVolt / 0.5 ms	0.7 (1.8) mVolt / 0.5 ms	0.9 (0.9) mVolt / 0.5 ms

**Figure 3:**
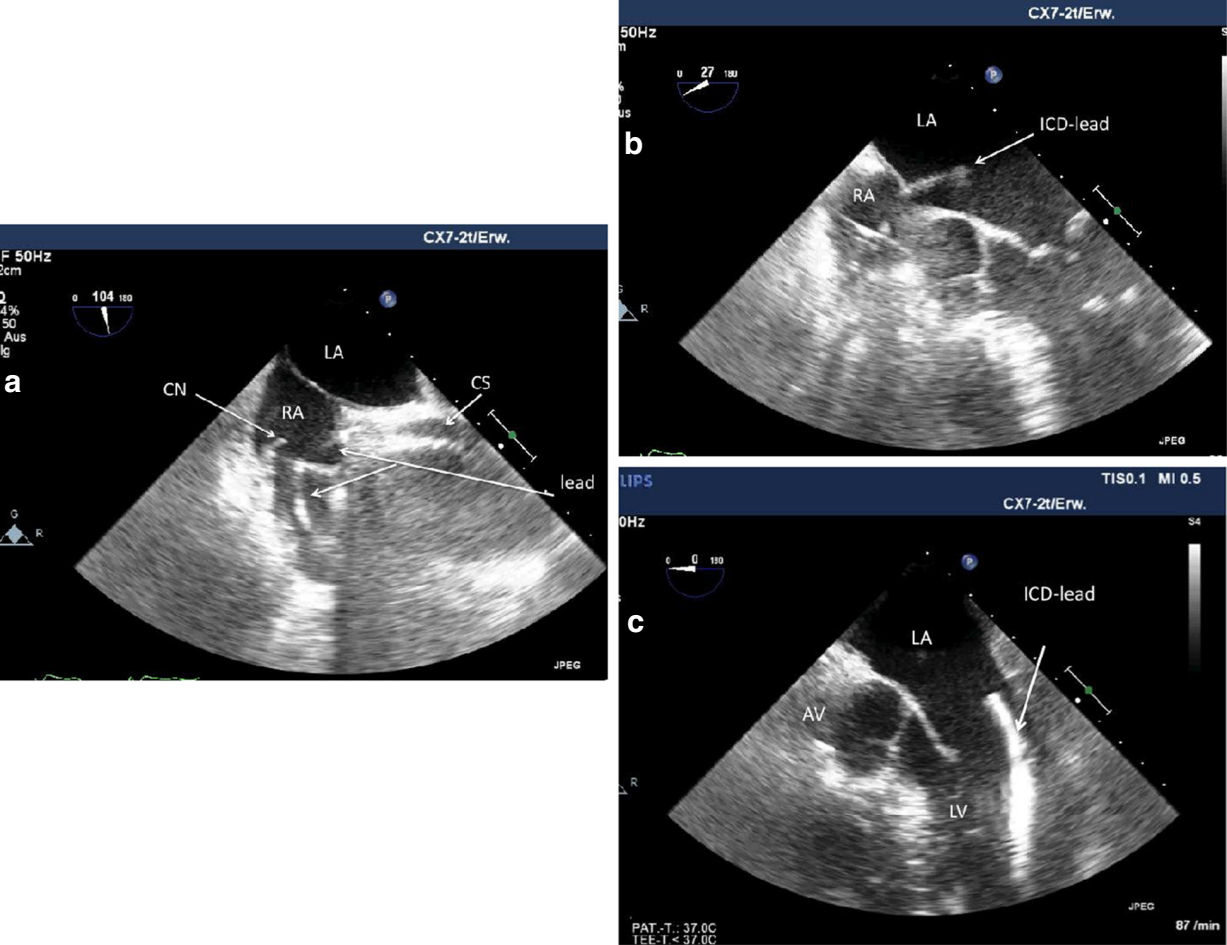
**Transesophageal echocardiographic recordings after the original implantation procedure.** The LV electrode (lead) is placed in the coronary sinus (CS), Chiari’s network (CN) present in the right atrium (RA) **(3 a)**; the ICD electrode (ICD lead) passes the atrial septum via the foramen ovale **(3 b)** into the left atrium (LA) and ventricular (LV) apex **(3 c)**.

One month later on follow-up, the patient reported a marked improvement of his exercise capacity (NYHA II). Electrode values remained stable. An unscheduled echocardiogramm (Philips iE33) revealed a marked reduction of left ventricular dimensions with an improved monoplane ejection fraction (51%). However, through the mitral valve leaflets a thick electrode proceeded into the apex of the left ventricle. An additional transesophageal echocardiographic examination demonstrated the coronary sinus (CS) electrode in place (Figure 3a) but a malposition of the RV electrode passing the patent foramen ovale (PFO) via an Eustachian valve into the left ventricle (3 b-d).

After studying the literature and informing the patient about possible consequences a revision procedure was performed. The RV electrode was easily removed from the left ventricle but could not be safely placed in the right heart without a sheat. A new electrode was advanced and fixed at the base of the right ventricle. Postprocedure chest x-ray (Figure 2c-d), ECG (Figure 1e-f), and measurements (Table 1) all documented good results. This time a postprocedure echocardiogramm showed an intact mitral valve, persistent good left performance, and the RV electrode within the right heart.

This was a clinical study and no experimental work. No ethical committee had to be involved. Everything happened in the compliance with the Helsinki Declaration.

## Discussion

Inadvertent placement of a transvenous RV electrode in the left ventricle has been described by several case reports and reviews [4,7]. Electrodes usually reach the left cavity either by direct arterial puncture, via a PFO [3,4,7] or atrial septal defect, or by perforation of the RV wall or interventricular septum [4,5,7,8]. Like the present case, passage of the PFO may be fascilated and induced by a prominent Eustachian valve [1]. During implantation electrode misplacement may be difficult to recognize in particular if the lead is inserted by posterioranterior fluoroscopic guidance [9,10]. Lateral projections during the operation should be used to ensure correct lead positioning. At the end of the procedure, electrode placement should be documented by antero-posterior and lateral takes [9,10].

In patients with right ventricular apical pacing postprocedure ECG is diagnostic in most patients revealing a RBBB with positive deflection in lead V1 and a negative deflection in lead I. Typically, RV pacing results in a LBBB with negative deflection in V1 and positive in I. This finding has a good sensitivity for malposition or perforation of the RV electrode [11]. However, patients with RBBB configurations after transvenous RV pacing require careful evaluation. In some cases, “safe” RBBB patterns may occur in epicardial pacing, septal pacing, and even in right apical pacing [11,12]. In CRT paced patients, ECG signs are not helpful because biventricular pacing (RV and LV lead simultaneously) usually results in an expected RBBB ECG pattern. An RV lead inadvertently placed in the LV may be identified by single testing under 12 lead ECG control, which is uncommon during routine postoperative follow-up. The frequency of LV misplacement in the CRT population remains unknown and is possibly underdiagnosed.

RV malplacement may be detected by postero-anterior and lateral chest radiographs in particular if the tip shows a posterior orientation [4,6,9]. However, diagnosis of RV malpositioning requires a high degree of suspicion [10]. In the present case, routine examination of chest x-ray (2a-b) was judged normal. By careful retrospective analysis, however, the RV lead curve is unusual and needs futher investigation. In the present case, diagnosis of malplacement was made by transthoracic echocardiography 4 weeks after the implantation. Instead of transesophageal echocardiography, chest CT scan would be an alternative diagnostic possibility [10].

Electrodes in the arterial circulation without adequate anticoagulation may cause cerebral and systemic embolism [4,7,13] or even left sided endocarditis and valve injury [4,14]. However, incidental diagnosis with asymptomatic course over years of follow-up is common [4,6,7,15]. Thrombembolic events may occur in up to 37% of patients [4,5,14]. The management of left ventricular malposition depends on three factors [4,7]: 1. the time from implantation of the misplaced lead, 2. the presence of thrombus on the lead documented by transesophageal echo, 3. the occurence of thrombembolic events. In an asymptomatic case like the present patient with recent implantation without thrombus or embolic event, simple traction of the electrode possibly accompanied by effective anticoagulation may be the best treatment option [4,7]. The unproven safety of extraction devices in the presence of left sided electrodes with the potential of thrombembolic complications has to be considered in more complicated patients with risk factors. Chronic situations with older electrodes especially in the presence of thrombus or recent embolism may be treated by permanent anticoagulation or even by open heart surgery [2,3,7,8].

## Conclusion

Inadvertent malposition of an RV lead in the left ventricle may be difficult to diagnose in patients with CRT devices. Lateral radiographic views during the operation should be used to ensure correct lead positioning. In biventricular stimulation, ECG criteria commonly used in RV stimulation are not applicable. In case of electrode malplacement, early operative revision seems to be the favorite strategy. There should be a low threshhold performing routine transthoracic echocardiography days or a few weeks after the CRT device implantation procedure.

## Consent

Written informed consent was obtained from the patient for publication of this case report and the accompanying images. A copy of the written consent is available by the Editor-in-Chief of the journal.

## Abbreviations

RV: Right ventricle; CRT: Cardiac resynchronisation therapy; LV: Left ventricle; EF: Ejection fraction; CRT-ICD: Cardiac resynchronisation therapy - implantable cardioverter defibrillator; NYHA: New York Heart Association; LBBB: Left bundle branch block; ECG: Electrocardiogram; RBBB: Right bundle branch block; TEE: Transesophageal echocardiogram; PFO: Patent foramen ovale.

## Competing interests

The authors declare that they have no competing interests.

## Authors’ contributions

RD conceived, designed and drafted the manuscript, assisted in the operation and was the consultant in charge of the patient’s care. UW was the principal operating cardiologist and collected various imaging data. MZ was primarily involved in the clinical and scientific discussion of the case. All three authors revised the manuscript and made intellectual contributions.
